# Glycemic variability and time in range among children with type 1 diabetes on insulin pump during the Covid-19 pandemic in Egypt; single center experience

**DOI:** 10.1186/s12902-023-01517-w

**Published:** 2023-11-27

**Authors:** Abeer Ahmed Abdel Maksoud, Nouran Yousef Salah, Safaa Alshraki Alsayed Ayoup

**Affiliations:** 1 Pediatrics Department, Faculty of Medicine, Ain Shams University, 25 Korash Street, Nasr City, Cairo, Egypt; 2 Al Rahmaneya Hospital, El Behera, Egypt

**Keywords:** Covid-19, Glycemic variability, Time in range, Children, T1DM

## Abstract

**Background:**

Covid-19 has impacted the lives of individuals worldwide especially those with chronic illnesses. Children with type 1 diabetes (T1DM) are at risk of glycemic deterioration during the Covid-19 pandemic. However, some studies reported glycemic improvement in these children during the pandemic.

**Aim:**

To assess the impact of Covid-19 on glycemic control and acute complications among children with T1DM on insulin pump in Egypt.

**Methodology:**

Forty-two children with T1DM on insulin pump for at least 1 year were assessed during the period from June 2020 to May 2021 for insulin requirements, insulin-pump problems, frequency of diabetic-ketoacidosis (DKA), hypoglycemia and HbA1C. Continuous-glucose monitoring was done using Medtronic i-pro device for 5 days. Data were compared to those obtained from the patients’ medical records 1 year previously.

**Result:**

Upon comparing data during Covid-19 pandemic with previous data from 12–24 months before Covid-19, there was a significant small increase in the mean total daily insulin dose from 0.83 ± 0.28 to 0.88 ± 0.30 U/kg/day with a similar small increase in the mean basal percentage from 51.19 ± 3.46 to 52.74 ± 4.31. Interestingly, the median time in range showed small increase from 53 (IQR 47–61) to 57.0 (IQR 51–73), the mean coefficient of variation showed small decrease from 42.10 ± 9.90 to 38.20 ± 8.12 and the mean HbA1C significantly decreased from 8.8 ± 1.3 (72.31 ± 16.78 mmol/ml) to 7.8 ± 1.2 mg/dl (61.31 ± 16.62 mmol/mol). Twenty-nine children (69%) had insulin-pump problems in the form of skin irritation (31%), skin infection (7.1%) and pump Set/Site occlusion (31%).

**Conclusion:**

No safety issues and overall glycemic improvement were reported among the children with T1DM on insulin pump therapy from this single center during the covid-19 pandemic.

## Introduction

Covid-19 has negatively influenced physical activity, dietary pattern and psychological status of individuals especially those with diabetes with increased feelings of stress and anxiety [1]. During the pandemic, hospitals and health care professionals were shifted to dealing with patients with Covid-19, which undermined the healthcare for other illnesses; all of which might have negatively influenced the glycemic control in people with diabetes [2].

In Pediatrics, data suggest that Covid-19 outcomes and prognosis in children and adolescents with T1DM are similar to their peers without diabetes and consistently milder than adults with diabetes [3].

The use of continuous glucose monitoring (CGM) system and telemedicine has allowed healthcare professionals to remotely monitor changes in glycemic control during the Covid-19 pandemic [4]. Children with T1DM wearing insulin pumps were found to have better glycemic control and better coping with stress and anxiety caused by the pandemic, with fewer hyperglycemic episodes than those on multiple daily injections (MDI) [5].

Although the impact of Covid-19 on adults with diabetes has been reported in various studies [6–8], there is paucity and discrepancy of literature about its impact on children with T1DM. Hence, this study aimed to assess the impact of COVID-19 on glycemic control among children with T1DM on insulin pump in Egypt and to explore it’s relation to various clinico-demographic parameters and acute diabetes complications.

## Methodology

### Study design and ethical considerations

This real life cohort study included forty two children and adolescent with T1DM on insulin pump for at least 1 year, aged 6–18 years old. T1DM was defined according to the criteria of the ISPAD 2018 [9]. Exclusion criteria included children with other types of diabetes (e.g. T2DM, maturity onset diabetes of youth, secondary diabetes), patients with comorbid diseases e.g. autoimmune diseases, psychiatric disorders, cardiac, neurological and hepatic disorders. Participants were enrolled from the regular attendees of the Pediatrics and Adolescent Diabetes Unit (PADU), faculty of medicine, Ain-shams University during the period from June 2020 to May 2021.

Ethical approval was obtained from the Research Ethics Committee of Faculty of Medicine, Ain Shams University (FMASU REC) with an approval number MS 496/2020. A written informed consent was obtained from each patient and/or their legal guardians prior to enrollment in the study.

Advice for study design and sample size was taken from the public health department, Faculty of Medicine, Ain Shams University. Sample size was calculated using G*power program, setting the type-1 error (α) at 0.05 and the power (1- β) at 0.8. Result from previous study [10], showed that the mean glucose before Covid-19 pandemic was 170 ± 45 mg/dl while during the pandemic was 160 ± 40 mg/dl, with an assumed correlation of 0.75 between pre and during glucose values. Calculation according to these values produced a sample size of at least 38 cases to be representative.

### Study procedures

Detailed medical history was taken with special emphasis on demographic data, age at onset of diabetes, diabetes duration, insulin therapy and history of acute metabolic complications (frequency of DKA in the last year and hypoglycemia and nocturnal hypoglycemia in the last week). History of suggestive of any complications related to insulin pumps use was taken as irritation, skin infection or pump Set/Site occlusion. Insulin therapy was assessed including basal rate, insulin carbohydrate ratio, insulin sensitivity score and total daily insulin requirements. The frequency and method of health care access and availability (delivery and cost) of insulin pump supplies (including infusion sets, reservoirs and cartridges, batteries, prep wipes, tape, and liquid adhesive) and glucose strips were sought. Assessment of any complications related to insulin pumps as pump Set/Site occlusion or skin infection was done. History was collected during regular clinic visits as well as Telemedicine visits phone calls, video calls, whatsapp group and telemonitoring.

Thorough clinical examination was done laying stress on anthropometric measures including weight in kilograms (Kg), height in centimeters (cm) and body mass index (BMI) in kg/m2 with plotting them on the age and gender standard percentiles [11].

Mean fraction-C of glycosylated hemoglobin (HbA1C) in the last year prior to the study was assessed using D-10 BioRad, France [12].

### Continuous glucose monitoring

Medtronic iPro2 Recorder continuous glucose monitoring system (CGM) was used through insertion of a glucose oxidase-based sensor in the subcutaneous area of the abdomen. The system recorded the glucose readings over 24 h continuously for 5 days. All candidates were instructed to follow their usual diet and insulin regimen. Calibration of the sensor with the glucometer was done after 2 h, 8 h and 12 h, respectively, to assure accuracy. The recorded data was obtained and downloaded using Medtronic Diabetes, CareLink software. Nocturnal hypoglycemia, hyperglycemia, average glucose and time in range (TIR) were obtained for each participant. TIR is defined as time spent between 70–180 mg/dL (3.9–10 mmol/L), hyperglycemia is defined as CGMS reading > 180mg/dl (10 mmol/L); while hypoglycemia is defined as CGMS reading < 70mg/dl (3.9 mmol/L) [13].

Glycemic Variability (GV) is the degree of fluctuation in blood glucose values over a given time period. In the short term, these oscillations can be described with intraday variability metrics. Currently, the coefficient of variation (%CV) is considered the metric of choice to describe intraday GV. International consensus on CGM recommends classifying values of %CV ≥ 36 as high GV and patients with these values as unstable [14].

- All clinical, laboratory and CGM collected data for assessment during the Covid-19 pandemic were recorded in the period from June 2020 to May 2021. They were compared with previous participant’s data in the period from June 2018 to May 2019 obtained from the patient and the caregiver and the patients’ medical records.

### Statistical analysis

Data were collected, revised, coded and entered to the Statistical Package for Social Science (IBM SPSS) version 23. The quantitative data were presented as mean, standard deviations and ranges when their distribution found parametric and median with inter-quartile range (IQR) when their distribution found non parametric. Also qualitative variables were presented as number and percentages.

The comparison between two groups regarding qualitative data were done by using *Chi-square test* and *Fisher exact test* instead of Chi-square test when the expected count in any cell found less than 5.

The comparison between two independent groups with quantitative data and non-parametric distribution was done by using *Mann-Whitney test* while the comparison between more than two groups was done by using *Kruskall-Wallis test*. Spearman correlation coefficients were used to assess the correlation between two quantitative parameters in the same group. The confidence interval was set to 95% and the margin of error accepted was set to 5%. So, the *p*-value was considered significant when *p*-value is < 0.05.

## Results

The mean age of the studied children with T1DM was 12.31 ± 3.28 years. They were 15 males and 27 females with a male to female ratio of 1: 1.8. The mean duration of insulin pump wearing at the study start was 39.23 months, range 24–52. Their mean insulin requirements during the Covid-19 pandemic were 0.88 ± 0.30, range 0.5- 1.8 with a median ICR of 15, range 8–50 and ISF of 52.5, range 20–150. None of the studied children reported problems with the availability of pump supplies, whereas twenty nine children (69%) had insulin pump problems in the form of skin irritation (31%), skin infection (7.1%) and pump Set/Site occlusion (31%). The clinico-demographic data of the studied children with T1DM on insulin pump during COVID-19 pandemic are listed in Table 1.

**Table 1:** Clinico-demographic of the studied children with T1DM on insulin pump during COVID-19 pandemic

	**Children with T1DM on insulin pump during COVID-19** **No. = 42**
Age (Year)	
Mean ± SD	12.31 ± 3.28
Range	7–18
Gender	
Female	27 (64.3%)
Male	15 (35.7%)
Duration of diabetes (Years)	
Mean ± SD	5.40 ± 2.93
Range	2–13
Weight z-score	
Median (IQR)	-0.31 (-0.79–0.61)
Range	-1.5–2.28
Height z-score	
Median (IQR)	0.04 (-0.82–0.52)
Range	-2.3–2.31
BMI z-score	
Median (IQR)	-0.19 (-0.62–0.64)
Range	-2.49–2.89
Insulin dose (unit/Kg/day)	
Mean ± SD	0.88 ± 0.30
Range	0.5–1.8
Basal percentage (%)	
Mean ± SD	52.74 ± 4.31
Range	50–60
ICR	
Median (IQR)	15 (10–20)
Range	8–50
ISF	
Median (IQR)	52.5 (40–80)
Range	20–150
Insulin pump problems	
Positive	29 (69.0%)
Skin irritation	13 (31.0%)
Skin infection	3 (7.1%)
Pump obstruction	13 (31.0%)
Availability of insulin pump supplies	
Positive	42 (100.0%)
DKA / year	
Positive	6 (14.3%)
Frequency of hypoglycemia /week	
Median (IQR)	3 (2–3)
Range	0–7
Frequency of nocturnal hypoglycemia /week	
Median (IQR)	2 (1–3)
Range	1–4

*T1DM* Type 1 diabetes mellitus, *BMI* Body mass index, *ICR* Insulin to carbohydrate ratio, *ISF* Insulin sensitivity factor, *DKA* Diabetic ketoacidosis

Regarding CGM data, the median time in range spent was 57%, range 12–80, and the mean coefficient of variation was 38.2%, range 23.3–54.4, Table 2.

**Table 2:** Glycemic data of the studied children with T1DM on insulin pump during COVID-19

	**Children with T1DM on insulin pump during COVID-19** **No. = 42**
Maximum CGM reading (mg/dl)	
Mean ± SD	336.38 ± 44.58
Range	251–400
Maximum CGM reading (mmol/L)	
Mean ± SD	18.69 ± 44.58
Range	13.94 ± 22.22
Minimum CGM reading (mg/dl)	
Mean ± SD	50.71 ± 12.88
Range	40–89
Minimum CGM reading (mmol/L)	
Mean ± SD	2.82 ± 0.72
Range	2.22–4.94
Average CGM reading (mg/dl)	
Mean ± SD	163.02 ± 31.89
Range	105–265
Average CGM reading (mmol/L)	
Mean ± SD	9.06 ± 1.77
Range	5.83–14.72
Time above range (%)	
Median (IQR)	39 (30.5–45)
Range	10–86
< 25	8 (19.0%)
≥ 25	34 (81.0%)
Time in range (%)	
Median (IQR)	57.0 (51–73)
Range	12–80
< 70	24 (57.1%)
≥ 70	18 (42.9%)
Time below range (%)	
Median (IQR)	3 (1–11)
Range	0–16
< 5	18 (42.9%)
≥ 5	24 (57.1%)
Coefficient of variation (%)	
Mean ± SD	38.20 ± 8.12
Range	23.3–54.4
< 36	22 (47.6%)
≥ 36	20 (52.4%)
HbA1C (mg/dl)	
Mean ± SD	9.0 ± 1.2
Range	6.5–11
HbA1C (mmol/mol)	
Mean ± SD	75 ± 16.62
Range	48–97
< 7	14 (33.3%)
≥ 7	28 (66.7%)

*T1DM* Type 1 diabetes, *HbA1C* Glycated hemoglobin, *CGM* Continuous glucose monitoring system, *LDL* Low density lipoproteins, *HDL* High density lipoproteins, *UACR* Urinary albumin creatinine ratio

**Fig. 1:**
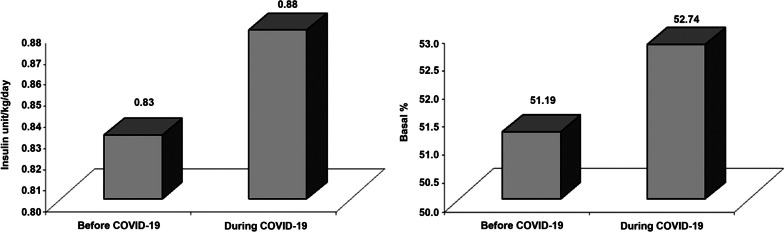
Daily insulin requirements and basal percentage among children with T1DM on insulin pump during and before Covid-19

**Fig. 2:**
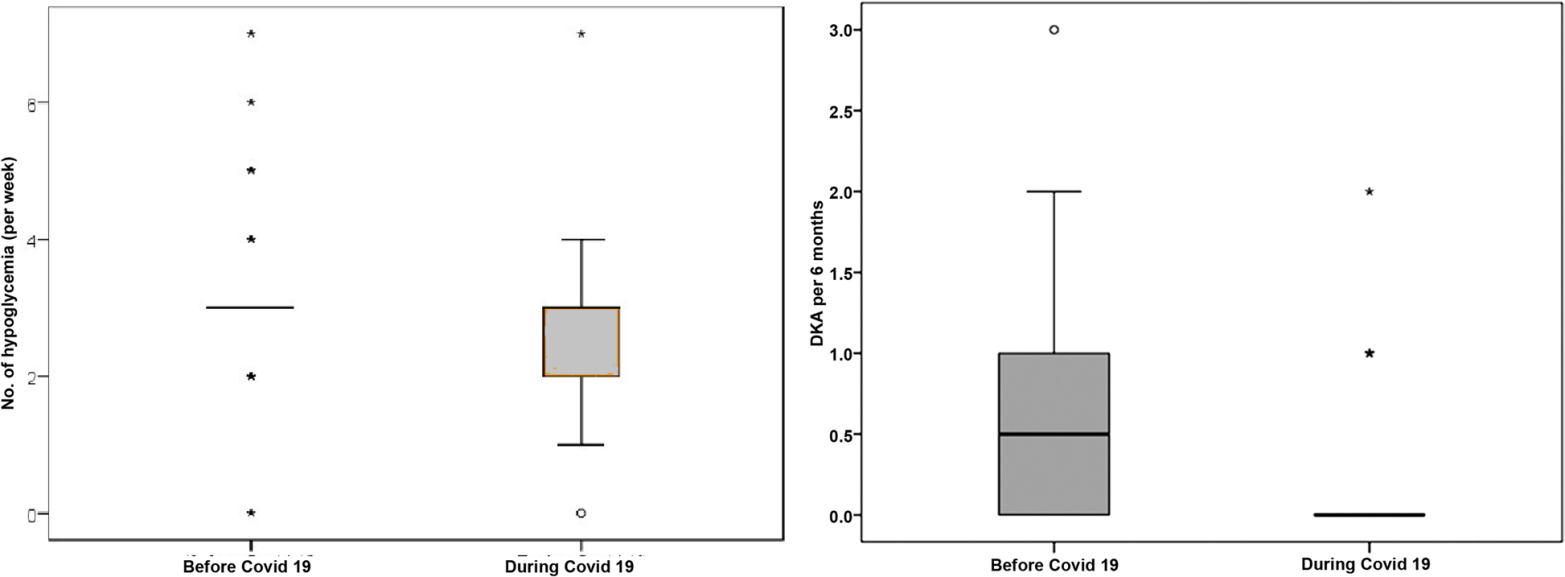
Comparison between the frequency of hypoglycemia and DKA during and before Covid-19 among children with T1DM on insulin pump

**Fig. 3:**
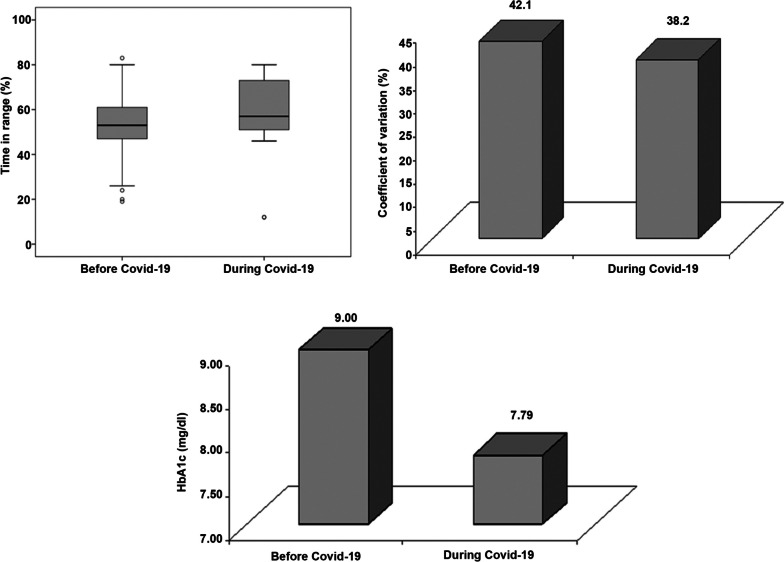
Glycemic parameters of the studied children with T1DM during and before Covid-19

**Fig. 4:**
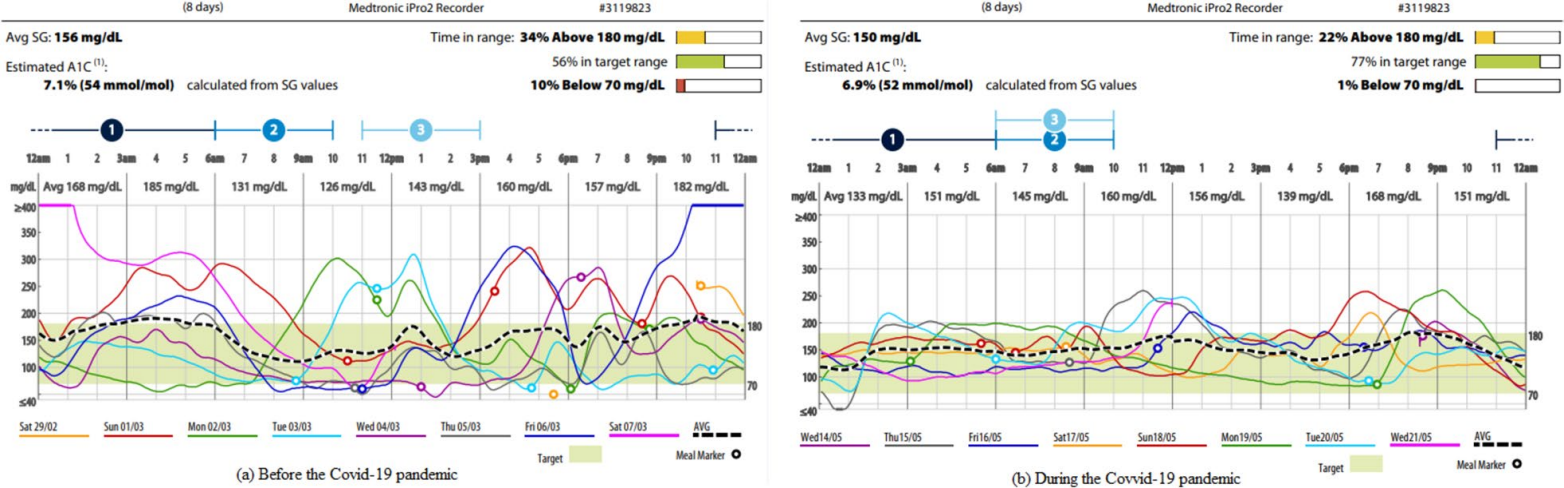
Overlay of continuous glucose monitoring record of male patient no.14 aged 11 years old before (**a**) and during (**b**) the Covid-19 pandemic

Upon comparing data during Covid-19 pandemic with previous data from 12–24 months before Covid-19, there was a significant small increase in the mean total daily insulin dose from 0.83 ± 0.28 to 0.88 ± 0.30 U/kg/day (*p* = 0.001) with a similar small increase in the mean basal percentage from 51.19 ± 3.46 to 52.74 ± 4.31 (*p* = 0.011), Fig. 1 and Table 3. Notably, the number of children with T1DM on insulin pump who developed DKA per year decreased significantly from 21 children before the Covid-19 (50%) to 6 children (14.3%) during the Covid-19 pandemic (*p* = 0.007), Fig. 2 and Table 3. Interestingly, small increase in the median time in range from 53 (IQR 47–61) to 57.0 (IQR 51–73), *p* = 0.009, small decrease in the mean coefficient of variation from 42.10 ± 9.90 to 38.20 ± 8.12, (*p* = 0.001) and significant decrease in the mean HbA1C from 8.8 ± 1.3 (72.31 ± 16.78 mmol/ml) to 7.8 ± 1.2 mg/dl (61.31 ± 16.62 mmol/mol), *p* = 0.001 were reported during the Covid-19 pandemic, Figs. 3 and 4 and Table 3.

**Table 3:** Comparison of the clinico-laboratory and CGM data of the studied children with T1DM on insulin pump before and during the Covid-19 pandemic

	**Before Covid-19**	**During Covid-19**	**Test value**	***P*-value**
	**No. = 42**	**No. = 42**		
Weight z-score				
Median (IQR)	-0.3 (-0.75–0.67)	-0.31 (-0.79–0.61)	-0.131^c^	0.896
Range	-1.45–2.26	-1.5–2.28		
Height z-score				
Median (IQR)	0.1 (-0.77–0.53)	0.04 (-0.82–0.52)	-1.857^c^	0.063
Range	-2.59–2.35	-2.3–2.31		
BMI z-score				
Median (IQR)	-0.03 (-0.69–0.69)	-0.19 (-0.62–0.64)	-1.369^c^	0.171
Range	-2.51–2.54	-2.49–2.89		
Insulin dose (unit/Kg/day)				
Mean ± SD	0.83 ± 0.28	0.88 ± 0.30	3.757^b^	**0.001**
Range	0.5–1.7	0.5–1.8		
Basal percentage (%)				
Mean ± SD	51.19 ± 3.46	52.74 ± 4.31	2.680^b^	**0.011**
Range	45–60	50–60		
ICR				
Median (IQR)	15 (10–20)	15 (10–20)	-1.300^c^	0.194
Range	8–30	8–50		
ISF				
Median (IQR)	57.5 (40–90)	52.5 (40–80)	-0.052^c^	0.959
Range	20–150	20–150		
Insulin pump problems				
Negative	17 (40.5%)	13 (31.0%)	0.830^a^	0.362
Positive	25 (59.5%)	29 (69.0%)		
DKA /year				
Positive	21 (50%)	6 (14.3%)	12.104^c^	**0.007**
Frequency of hypoglycemia/ week				
Median (IQR)	3 (3–3)	3 (2–3)	-2.858^a^	**0.004**
Range	0–7	0–7		
Frequency of nocturnal hypoglycemia/ week				
Median (IQR)	2 (2–4)	2 (1–3)	1.340	0.180
Range	1–5	1–4		
Time above range (%)				
Median (IQR)	41 (31–47)	39 (30.5–45)	0.506^c^	0.613
Range	13–75	10–86		
< 25	6 (14.3%)	8 (19.0%)	0.343^a^	0.558
≥ 25	36 (85.7%)	34 (81.0%)		
Time in range (%)				
Median (IQR)	53 (47–61)	57.0 (51–73)	2.105^c^	**0.035**
Range	19–83	12–80		
< 70	35 (83.3%)	24 (57.1%)	6.891^a^	**0.009**
≥ 70	7 (16.7%)	18 (42.9%)		
Time below range (%)				
Median (IQR)	6 (2–8)	3 (1–11)	0.330^c^	0.742
Range	0–24	0–16		
< 5	18 (42.9%)	18 (42.9%)	0.000^a^	1.000
≥ 5	24 (57.1%)	24 (57.1%)		
Coefficient of variation (%)				
Mean ± SD	42.10 ± 9.90	38.20 ± 8.12	-2.174^b^	**0.036**
Range	20.3–77.4	23.3–54.4		
< 36	8 (19.0%)	22 (47.6%)	10.163^a^	**0.001**
≥ 36	34 (81.0%)	20 (52.4%)		
HbA1C (mg/dl)				
Mean ± SD	8.8 ± 1.3	7.8 ± 1.2	4.263^b^	**0.001**
Range	6–11.5	4.9–11.3		
HbA1C (mmol/mol)				
Mean ± SD	72.31 ± 16.78	61.31 ± 16.62		
Range	42– 102	30–100		

*P*-value < 0.05: Significant

*T1DM* Type 1 diabetes mellitus, *BMI* Body mass index, *ICR* Insulin to carbohydrate ratio, *ISF* Insulin sensitivity factor, *DKA* Diabetic ketoacidosis, *CGM* Continuous glucose monitoring system, *HbA1C* Glycated hemoglobin

^a^Chi-square test

^b^Paired t- test

^c^Wilcoxon Rank test

Interestingly, CGM data showed a 12.68% increase in the time in range and 11.33% decrease in the coefficient of variation than the previous year, Table 4.

**Table 4:** Percentage of change of the studied parameters during the Covid-19 pandemic

	**Children with T1DM on insulin pump** **No. = 42**
BMI	
Median (IQR)	3.53 (1.40–5.14)
Range	-17.26–11.04
Insulin dose (unit/Kg/day)	
Median (IQR)	0.00 (0.00–14.29)
Range	-14.29–28.57
Basal %	
Median (IQR)	0.00 (0.00–0.00)
Range	0.00–33.33
ICR	
Median (IQR)	0.00 (0.00–0.00)
Range	-20.00–66.67
ISF	
Median (IQR)	0.00 (0.00–0.00)
Range	-16.67–40.00
Range	-100.00–250.00
HbA1C (mg/dl)	
Median (IQR)	-10.59 (-22.33–-1.74)
Range	-44.92–7.14
HbA1C (mmol/mol)	
Median (IQR)	-13.75 (-28.13- -1.96)
Range	65.12- 10.14
Time above range %	
Median (IQR)	-3.73 (-41.18–38.71)
Range	-64.71–315.38
Time in range %	
Median (IQR)	12.68 (-6.56–35.71)
Range	-75.51–284.21
Time above range %	
Median (IQR)	-9.82 (-83.33–120.00)
Range	-100.00–800.00
Coefficient of variation %	
Median (IQR)	-11.33 (-20.09–6.80)
Range	-60.34–46.80

*BMI* Body mass index, *ISF* Insulin sensitivity factor, *ICR* Insulin to carbohydrate ratio, *DKA* Diabetic ketoacidosis, *HbA1C* Glycatedhaemoglobin, *CGMS* Continous glucose monitoring system, *LDL* Low density lipoprotein, *HDL* High density lipoprotein, *UACR* Urine albumin creatinine ratio

## Discussion

Glycemic deterioration has been reported as a complication of Covid-19 in patients with impaired glucose regulation or diabetes mellitus [4]. Nevertheless, data about the impact of Covid-19 on glycemic control in children with T1DM is scarce; with no similar studies form Egypt. Two studies conducted in Italy have reported improved glycemic control early during the Covid-19 pandemic in adults with T1DM using flash glucose monitoring. They attributed this to slowing down their routine daily activities as these patients had stopped working routine and the absence of the usual daily stress levels [10, 15]. In contrast, Tao et al. reported glycemic deterioration during the Covid-19 pandemic, which they attributed to older age, less education, poor medication compliance and less self-monitoring blood glucose [16].

In the pediatric population, Wu et al. 2021 showed no deterioration in glycemic control in children and adolescents with T1DM in China during the Covid-19 pandemic [17]. However, Predieri et al. reported improvement of glycemic control in the form of decreased coefficient of variation and increased time in range in children with T1DM using CGM, in a cohort from Italy. They attributed this to the more stable life rhythm and more constant parental diabetes care during the pandemic [18]. This goes in concordance with the current study that found significant improvement in the HbA1C, time in range and coefficient of variation among the studied children with T1DM on insulin pump during the Covid-19 pandemic than the previous year. The improvements in control are likely reflecting increased supervision and perhaps attention by the parents of children with T1DM during this period especially when there was theoretical worry diabetes may worsen infection and the implementation of telemedicine visits monthly through phone calls with a 24 h whats-app group for emergencies.

In the current study, the number of children with T1DM on insulin pump who developed DKA per year was found to decrease from 21 children before the Covid-19 (50%) to 6 children (14.3%) during the covid-19 pandemic. However, the small sample size limits generalization of these results. This goes in line with a study from Saudi Arabia that showed that despite the marked increase in the overall DKA frequency and severity during than before the Covid-19 pandemic, there was a decrease (although nonsignificant) in the frequency of DKA from 97 (86.6%) to 65 (73.8%) and severe DKA from 20 (83.3%) to 16 (69.5%) among children with known T1DM [19]. In a German study comparing the frequency of DKA among children with diabetes before and during the covid-19 pandemic, they found significant increase in the overall frequency of DKA in 2020 than the same period of time in 2019. However, these changes were not so evident among those with known diabetes; seven children (17.4%) in 2020 compared to six children (10.9%) during the same period of time in 2019 [20]. The overall increase in the DKA rates could be attributed to the increase of the frequency of newly diagnosed DKA with the occurrence of the pandemic due to the virus itself and the decreased health care access and fear of hospital visits that occurred during the pandemic; while the decrease or stability in the frequency of DKA among those with known T1DM especially those using insulin pump could be attributed to the increased close monitoring by the caregivers to their children with diabetes owing to the increased time spent with them and the fear of Covid-19 related morbidity and mortality in this vulnerable population that was suggested with the start of the pandemic and the increased use of telemedicine especially among those on insulin pump.

The use of diabetes technology is rapidly increasing worldwide, with new technologies being introduced to the market continuously aiming to achieve better glycemic control by the patients and their caregivers [21]. The current study showed improvement of the glycemic parameters of the studied children with T1DM on insulin pump during the Covid-19 pandemic. This goes in line with results from similar studies relating the improvement of glycemic control during the Covid-19 pandemic to the use of diabetes technology and telemedicine [18, 19]. In a study by Gherbon and colleagues involving 122 Romanians with T1DM; 80 on MDI and 22 on insulin pump; those on MDI showed significant worsening of their blood glucose levels during the Covid-19 pandemic in comparison to those on insulin pump who showed improvement of their blood glucose levels which they attributed to delayed administration of insulin and inadequate insulin dosing by those on the MDI. In addition, insulin pump users were found to have improved response to stress and anxiety [5]. This could be attributed to the use of telemedicine and the beneficial effect of diabetes technology including continuous glucose monitoring.

### Strength and limitations

Although this study was the first to report the impact Covid-19 on glycemic control among children with T1DM in Egypt; it has some limitations; first it is a single center study including children who were already on insulin pump (only a small sample of the clinics children are on insulin pump) which may not represent all diabetes treatment facilities in Egypt. Moreover, the small sample size might undermine the ability to draw causal inferences. Hence, further prospective studies with larger samples comparing children on insulin pump and those on MDI are needed to explore the sustainability of the changes reported in glycemic control and their relation to acute and chronic diabetes complications and to evaluate the effectiveness and outcomes of telemedicine services used.

## Conclusion

No safety issues and overall glycemic improvement were reported among the children with T1DM on insulin pump therapy from this single center during the covid-19 pandemic.

## Data Availability

Data will be available from the corresponding author upon request.

## References

[1] Ingram J, Maciejewski G, Hand CJ (2020). Changes in diet, sleep, and physical activity are associated with differences in negative mood during COVID-19 lockdown. Front Psychol.

[2] Fernández E, Cortazar A, Bellido V (2020). Impact of COVID-19 lockdown on glycemic control in patients with type 1 diabetes. Diabetes Res Clin Pract.

[3] Cardona-Hernandez R, Cherubini V, Iafusco D, Schiaffini R, Luo X, Maahs DM (2021). Children and youth with diabetes are not at increased risk for hospitalization due to COVID-19. Pediatr Diabetes.

[4] Capaldo B, Annuzzi G, Creanza A, Giglio C, De Angelis R, Lupoli R, Masulli M, Riccardi G, Rivellese AA, Bozzetto L (2020). Blood glucose control during lockdown for COVID-19: CGM metrics in Italian adults with type 1 diabetes. Diabetes Care.

[5] Gherbon A, Frandes M, Timar R, Timar B (2022). The impact of COVID-19 lockdown on glycemic balance in Romanian patients with type 1 diabetes mellitus. Diabetes Metab Syndr Obes.

[6] Zhou J, Tan J (2020). Diabetes patients with COVID-19 need better blood glucose management in Wuhan, China. Metabolism.

[7] Huang I, Lim MA, Pranata R (2020). Diabetes mellitus is associated with increased mortality and severity of disease in COVID-19 pneumonia–a systematic review, meta-analysis, and meta-regression. Diabetes Metab Syndr.

[8] Tornese G, Ceconi V, Monasta L, Carletti C, Faleschini E, Barbi E (2020). Glycemic control in type 1 diabetes mellitus during COVID-19 quarantine and the role of in-home physical activity. Diabetes Technol Ther.

[9] Mayer-Davis EJ, Kahkoska AR, Jefferies C, Dabelea D, Balde N, Gong CX, Aschner P, Craig ME (2018). ISPAD Clinical Practice Consensus Guidelines 2018: definition, epidemiology, and classification of diabetes in children and adolescents. Pediatr Diabetes.

[10] Bonora BM, Boscari F, Avogaro A, Bruttomesso D, Fadini GP (2020). Glycaemic control among people with type 1 diabetes during lockdown for the SARS-CoV-2 outbreak in Italy. Diabetes Ther.

[11] World Health Organization. Department of Nutrition for Health and Development. WHO child growth standards. Length/height-for-age, weight-for-age, weight-for-length, weight-for-height, and body mass index-for-age. Methods and development. Geneva: WHO press; World Health Organization; 2006. pp. 301–304.

[12] Goldstein DE, Little RR, Wiedmeyer HM, England JD, McKenzie EM (1986). Glycated hemoglobin: methodologies and clinical applications. Clin Chem.

[13] Battelino T, Danne T, Bergenstal RM, Amiel SA, Beck R, Biester T, Bosi E, Buckingham BA, Cefalu WT, Close KL, Cobelli C (2019). Clinical targets for continuous glucose monitoring data interpretation: recommendations from the international consensus on time in range. Diabetes Care.

[14] Lu J, Ma X, Zhang L, Mo Y, Lu W, Zhu W, Bao Y, Jia W, Zhou J (2020). Glycemic variability modifies the relationship between time in range and hemoglobin A1c estimated from continuous glucose monitoring: a preliminary study. Diabetes Res Clin Pract.

[15] Pla B, Arranz A, Knott C, Sampedro M, Jiménez S, Hernando I, Marazuela M (2020). Impact of COVID-19 lockdown on glycemic control in adults with type 1 diabetes mellitus. J Endocr Soc.

[16] Tao J, Gao L, Liu Q, Dong K, Huang J, Peng X, Yang Y, Wang H, Yu X (2020). Factors contributing to glycemic control in diabetes mellitus patients complying with home quarantine during the coronavirus disease 2019 (COVID-19) epidemic. Diabetes Res Clin Pract.

[17] Wu X, Luo S, Zheng X, Ding Y, Wang S, Ling P, Yue T, Xu W, Yan J, Weng J (2021). Glycemic control in children and teenagers with type 1 diabetes around lockdown for COVID-19: a continuous glucose monitoring*-based observational study. J Diabetes Investig.

[18] Predieri B, Leo F, Candia F, Lucaccioni L, Madeo SF, Pugliese M, Vivaccia V, Bruzzi P, Iughetti L (2020). Glycemic control improvement in Italian children and adolescents with type 1 diabetes followed through telemedicine during lockdown due to the COVID-19 pandemic. Front Endocrinol.

[19] Alaqeel A, Aljuraibah F, Alsuhaibani M, Huneif M, Alsaheel A, Dubayee M, Alsaedi A, Bakkar A, Alnahari A, Taha A, Alharbi K, Alanazi Y, Almadhi S, Khalifah R (2021). The impact of COVID-19 pandemic lockdown on the incidence of new-onset type 1 diabetes and ketoacidosis among Saudi children. Front Endocrinol.

[20] Loh C, Weihe P, Kuplin N, Placzek K, Weihrauch-Blüher S (2021). Diabetic ketoacidosis in pediatric patients with type 1- and type 2 diabetes during the COVID-19 pandemic. Metabolism.

[21] American Diabetes Association. Diabetes and Coronavirus (COVID-19). ADA; 2020. https://www.diabetes.org/coronavirus-covid-19. Accessed 1 Jan 2022.

